# MYB transcription factor isolated from *Raphanus sativus* enhances anthocyanin accumulation in chrysanthemum cultivars

**DOI:** 10.1007/s13205-016-0396-8

**Published:** 2016-02-23

**Authors:** Eun Sun Kee, Aung Htay Naing, Sun Hyung Lim, Jeung Sul Han, Chang Kil Kim

**Affiliations:** 1 Department of Horticultural Science, Kyungpook National University, Daegu, 702-701 Korea; 2 National Academy of Agricultural Science, Rural Development Administration, Suwon, 441-707 Korea

**Keywords:** Anthocyanin, RT-PCR, Spectrophotometer, Transcription factor

## Abstract

A MYB transcription factor gene, *RsMYB1*, from radish was introduced into the chrysanthemum cultivars ‘Peach ND’, ‘Peach Red’, and ‘Vivid Scarlet’ under the control of the cauliflower mosaic virus 35S promoter. Presence of *RsMYB1* in transgenic lines was confirmed using polymerase chain reaction (PCR). Results of reverse-transcription-PCR analysis revealed that the expression of *RsMYB1* was stable in all transgenic lines and could enhance the expression levels of three key biosynthetic genes (*F3H*, *DFR*, and *ANS*) involved in anthocyanin production. Accordingly, anthocyanin content was significantly higher in transgenic lines than in control of all the cultivars, although the increasement was not visually observed in any of the transgenic lines. Therefore, these results demonstrate that *RsMYB1* has potential to enhance anthocyanin content in the chrysanthemums.

## Introduction

*Chrysanthemum morifolium* Ramat. is one of the most important floricultural crops that occupies a prominent position in flower markets worldwide. In 2012, its cultivation area was 31 % of the total cut-flower cultivation area (1724 ha) in Korea (Ministry of Agriculture, Food, and Rural Affairs, 2012), and it has been increasing on a yearly basis. In summer, the heat stress due to elevated temperatures causes degradation of anthocyanin accumulation in flower petals, leading to low flower quality that causes economic loss in chrysanthemum fields (Eun et al. 2008).

Temperature has been considered as an important factor that influences anthocyanin biosynthesis in various plants, such as apple (Ubi et al. 2006; Lin-Wang et al. 2011), grape (Yamane et al. 2006), petunia (Shvarts et al. 1997), red orange (Lo Piero et al. 2005), and rose (Dela et al. 2003). Elevated temperatures decrease the accumulation of anthocyanin. Huh et al. (2008) claimed that flower coloration was observed in red chrysanthemum cultivars when they were grown at an elevated temperature (35 °C).

Genetic engineering with MYB transcription factors has been used to enhance anthocyanin accumulation in several plant species, since MYB transcription factors up-regulated anthocyanin biosynthesis in tobacco, petunia, apple, rose, and lily (Espley et al. 2007; Lin-Wang et al. 2010; Pattanaik et al. 2010; Quattrocchio et al. 2006; Yamagishi et al. 2010). Pattanaik et al. (2010) reported that overexpression of MYB (NtAn2) in tobacco enhanced anthocyanin accumulation and expression levels of chalcone synthase (*CHS*), chalcone isomerase (*CHI*), flavanone 3-hydroxylase (*F3H*), dihydroflavonol 4-reductase (*DFR*), and anthocyanidin synthase (*ANS*) genes. In apples, red fruit color is controlled by MdMYB10 (Espley et al. 2007). The purple phenotype of sweet potato is also strongly associated with IbMYB1, which induces all anthocyanin biosynthetic genes (Mano et al. 2007). LhMYB6 and LhMYB12 increase the expression levels of anthocyanin biosynthetic genes and determine the accumulation of anthocyanin (Yamagishi et al. 2010). Radish MYB transcription factor belongs to the R2R3-MYB transcription factor family and is highly expressed in the skin and flesh of three radish cultivars (‘Seo Ho’, ‘Man Tang Hong’, and ‘Hong Feng No.1’); strong anthocyanin accumulation has been observed in the radish cultivars (Park et al. 2011). Expression of the MYB transcription factor in petunia enhanced the expression level of anthocyanin biosynthetic genes (*CHI*, *CHS*, *F3H*, *DFR*, and *ANS*) as well as anthocyanin accumulation in our previous studies. Since the main anthocyanin biosynthesis pathway in chrysanthemum is the cyanidin-based pathway, *CHS*, *CHI*, *F3H*, *DFR*, and *ANS* are considered as the five key biosynthetic genes responsible for anthocyanin accumulation in chrysanthemums. Thus, it is quite interesting to investigate the role of *RsMYB1* in anthocyanin accumulation in chrysanthemum.

In this study, we used three different red chrysanthemum cultivars that have been observed to show coloration in summer. *RsMYB1* was introduced into the cultivars by using *Agrobacterium*-mediated transformation. Expression levels of anthocyanin biosynthetic genes regulated by *RsMYB* and associated anthocyanin accumulation were analyzed.

## Materials and methods

Three red chrysanthemum cultivars (*C. morifolium* Ramat.), namely, ‘Peach ND’, ‘Peach Red’, and ‘Vivid scarlet’, were obtained from Gumi Research Station. The cultivars were then proliferated in vitro, according to the method described by Naing et al. (2015a). Briefly, shoot tips with about 1 cm^2^ in size were cultured on Murashige and Skoog medium (MS; Duchefa Biochemie, Netherlands) containing 3 % (w/v) sucrose, 0.8 % plant agar, and 0.01 % activated charcoal. Then, the cultures were maintained at 25 ± 2 °C and a 16-h photoperiod (37 μmol m^−2^ s^−1^)

### Plasmid construction and transformation

In this study, *Agrobacterium tumefaciens* strain C58C1 carrying a binary vector pB7WG2D with *RsMYB1* isolated from radish (*Raphanus sativus* L.) was used. Rs*MYB1* was placed under the control of the cauliflower mosaic virus 35S promoter. In addition, the vector contained the gene *bar* for resistance to phosphinothricin (PPT) in transgenic plants.

Prior to transformation, infection solution of *Agrobacterium* strain harboring the binary vector pB7WG2D were cultured as described by Naing et al. (2014), and transformation was performed according to protocol described by Naing et al. (2016). Briefly, 100 leaf segments (5 cm^2^) excised from in vitro plants of the three different cultivars were incubated in the *Agrobacterium* infection solution. The leaf segments were cultured on an MS co-cultivation medium with 0.5 mg L^−1^ of BA and 0.5 mg L^−1^ of NAA, 3 % sucrose, and 3 g L^−1^ of Gelrite (pH 5.8) and placed in the dark at 25 ± 2 °C for 3 days. Then, the leaf segments were cultured on the same medium containing 125 mg L^−1^ Clavamox (Zoetis, India) and placed in the dark at 25 ± 2 °C for 7 days. The leaf segments were further cultured on the same medium containing 1 mg L^−1^ of PPT and 125 mg L^−1^ of Clavamox under a 16-h photoperiod (37 μmol m^−2^ s^−1^). They were transferred to a fresh medium every 3 weeks to suppress *Agrobacterium* growth. After 6 weeks, green shoots resistant to PPT were collected and transferred to plant growth regulator-free MS medium with 1 mg L^−1^ PPT and 125 mg L^−1^ of Clavamox to assess plant growth. PPT-resistant plants that were 4–5 cm in size were transplanted to a tray with vermiculite soil and acclimatized in a greenhouse at 25 °C. The plantlets were then transferred to pots filled with peat soil and placed in the greenhouse.

### DNA extraction and polymerase chain reaction

Genomic DNA was extracted from young leaves of 6-week-old plants selected using 1 mg L^−1^ PPT with the RBC HiYield™ Genomic DNA Mini Kit (Real Biotech Corporation, Taiwan), according to manufacturer’s instructions. Genomic DNA was then amplified using polymerase chain reaction (PCR) with specific primers and PCR conditions mentioned in Table 1. To detect presence of *RsMYB1* and *bar*, amplified DNA was observed under UV (UVITEC Cambridge, UK) irradiation after electrophoresis for 30 min using 2 % agarose gel and staining with ethidium bromide.

**Table 1:** Primers and PCR conditions used in this study

Gene	Primer	Size (bp)	PCR condition
*RsMYB1*	F-ATG GAG GGT TCG TCC AAA GG	700	98 °C for 30 s, followed by 25 cycles including 10 min at 98 °C, 1 min at 98 °C and 1 min at 72 °C, and final elongation step at 72 °C for 1 min
	R-GAA ACA CTA ATC AAA TTA CAC AGT CTC TCC		
*Bar*	F-GGT CTG CAC CAT CGT	496	95 °C for 2 min, followed by 30 cycles including 20 s at 95 °C, 30 s at 55 °C and 30 s at 72 °C, and final elongation step at 72 °C for 5 min
	R-TCA GAT TTC GGT GAC GGG CA		
*CHS*	F-CAA CGG TTT TCT CCA TTA GGT	299	95 °C for 2 min, followed by 30 cycles including 20 s at 95 °C, 40 s at 57 °C and 30 s at 72 °C, and final elongation step at 72 °C for 5 min
	R-GAG GAC CAC GGT TTC GAC		
*CHI*	F-TGG TGC AAC CAT TGA CAA GT	300	95 °C for 2 min, followed by 30 cycles including 20 s at 95 °C, 40 s at 55.6 °C and 30 s at 72 °C, and final elongation step at 72 °C for 5 min
	R-AAA TTT GGT TCA GCA TCT GTA GTT		
*F3H*	F-ACC CGG TTC GTC CGT GAT GAG G	804	95 °C for 2 min, followed by 30 cycles including 20 s at 95 °C, 40 s at 64 °C and 30 s at 72 °C, and final elongation step at 72 °C for 5 min
	R-TGC CTG GTG GTC CGC ATT CT		
*DFR*	F-ATG AAA GAA GAC TCA CCA GCC A	1048	95 °C for 2 min, followed by 30 cycles including 20 s at 95 °C, 40 s at 59 °C and 30 s at 72 °C, and final elongation step at 72 °C for 5 min
	R-CTT CGT GAG TGT CCG CCT TT		
*ANS*	F-ATA CAT CCG AAC ACA AGA TG	432	95 °C for 2 min, followed by 30 cycles including 20 s at 95 °C, 40 s at 60 °C and 30 s at 72 °C, and final elongation step at 72 °C for 5 min
	R-AAT CGC TAG GTG TCG AGG GCC		
*Actin*	F-ACA ACG TTT TAC AAT GAG CTT CG	196	95 °C for 2 min, followed by 30 cycles including 20 s at 95 °C, 40 s at 57 °C and 30 s at 72 °C, and final elongation step at 72 °C for 5 min
	R-CCG TTC AGC AGT TGT AGT AA		

### RNA extraction and reverse transcription-PCR

To evaluate the expression level of *RsMYB1* and anthocyanin biosynthetic genes in the transgenic lines already confirmed using PCR, RNA of each line was extracted for reverse transcription (RT)-PCR analysis. Prior to RNA extraction, all equipment, including reagent bottles, were cleaned using RNA eraser (MP Bio, USA). Total RNA was isolated from 100 mg of leaf tissue of the transgenic and wild type chrysanthemum plants by using TRI Reagent™ Solution (Ambion, USA), according to the manufacturer’s instructions. Complementary DNA (cDNA) was synthesized from 100 ng of total RNA by using the High Capacity cDNA Reverse Transcription Kit (Applied Biosystems, USA), according to the manufacturer’s protocol. Primers and PCR conditions for *RsMYB1* remained unchanged, and those for the biosynthetic genes (*CHI*, *CHS*, *F3H*, *DFR*, and *ANS*) are listed in Table 1; *Actin* was used as the internal control. PCR products were observed under UV (UVITEC Cambridge, UK) irradiation after electrophoresis for 30 min using 2 % agarose gel and staining with ethidium bromide.

### Analysis of anthocyanin content

Total anthocyanin contents of the transgenic lines and control plants were analyzed according to the protocol used by Naing et al. (2015b), with some modifications. Briefly, approximately 500 mg of leaf materials excised from the plants grown in the greenhouse was crushed, and pigments were extracted with 5 mL of distilled water. They were then incubated overnight at 4 °C by adding 5 mL of 1 % (w/v) hydrochloric acid in methanol solution. The supernatant was collected after centrifugation at 3000 rpm for 20 min and transferred to a 2 mL collection tube. Absorbance was measured at 430–630 nm by using a spectrophotometer (U-2800; Hitachi, Tokyo, Japan). Anthocyanin content of each sample was calculated according to the method reported by Sung et al. (2013).

### Statistical analysis

For measurement of anthocyanin content, five samples from each plant (wild type and two independent transgenic lines) of the three chrysanthemum cultivars were detected. Data were analyzed using Duncan Multiple Range Test (DMRT) (*p* > 0.05).

## Results

### Generation of chrysanthemum transgenic plants that express *RsMYB1*

**Fig. 1:**
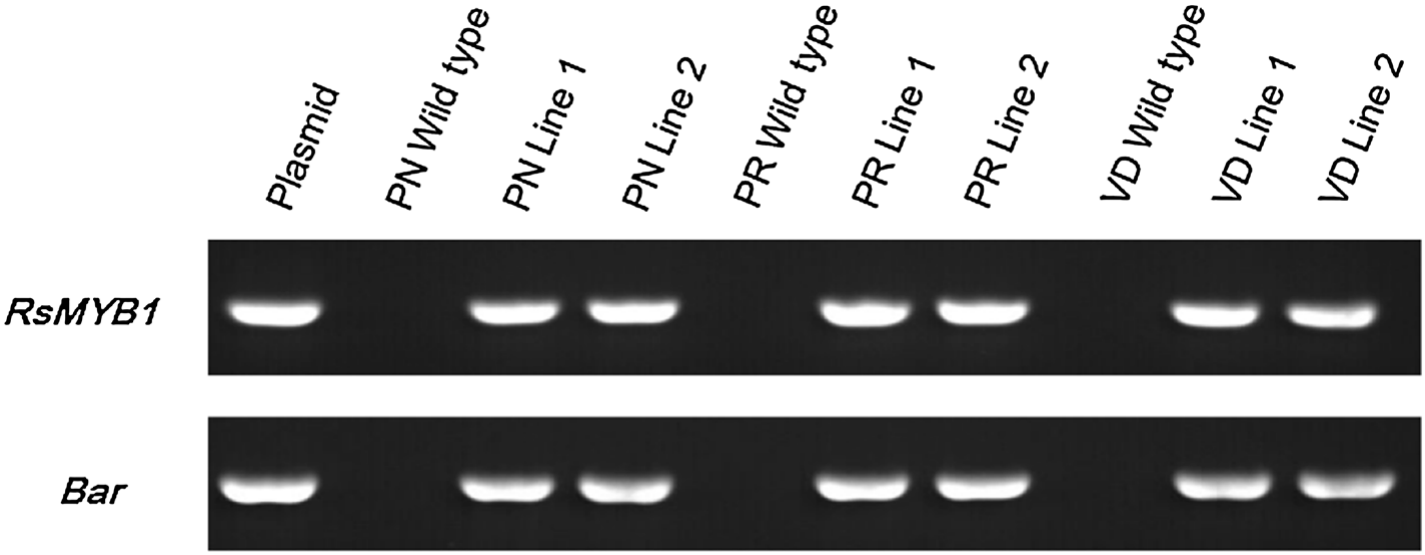
PCR detection of selection marker (*bar*), target (*RsMYB1*) genes in transgenic chrysanthemum (cvs. Peach ND (PN), Peach Red (PR), and Vivid Scarlet (VD) *lines* *1* and *2*

To investigate whether *RsMYB1* could enhance the expression level of biosynthetic genes responsible for anthocyanin biosynthesis, we produced two independent transgenic lines from the cultivars ‘Peach ND’, ‘Peach Red’, and ‘Vivid Scarlet’, in which the presence of the selection marker and transgene were detected using PCR (Fig. 1). Interestingly, enhanced anthocyanin expression was not observed in the transgenic plants of the cultivars. Thus, it was necessary to investigate the expression levels of the transgene and anthocyanin biosynthetic genes.

### Expression analysis of *RsMYB1* and anthocyanin biosynthetic genes

**Fig. 2:**
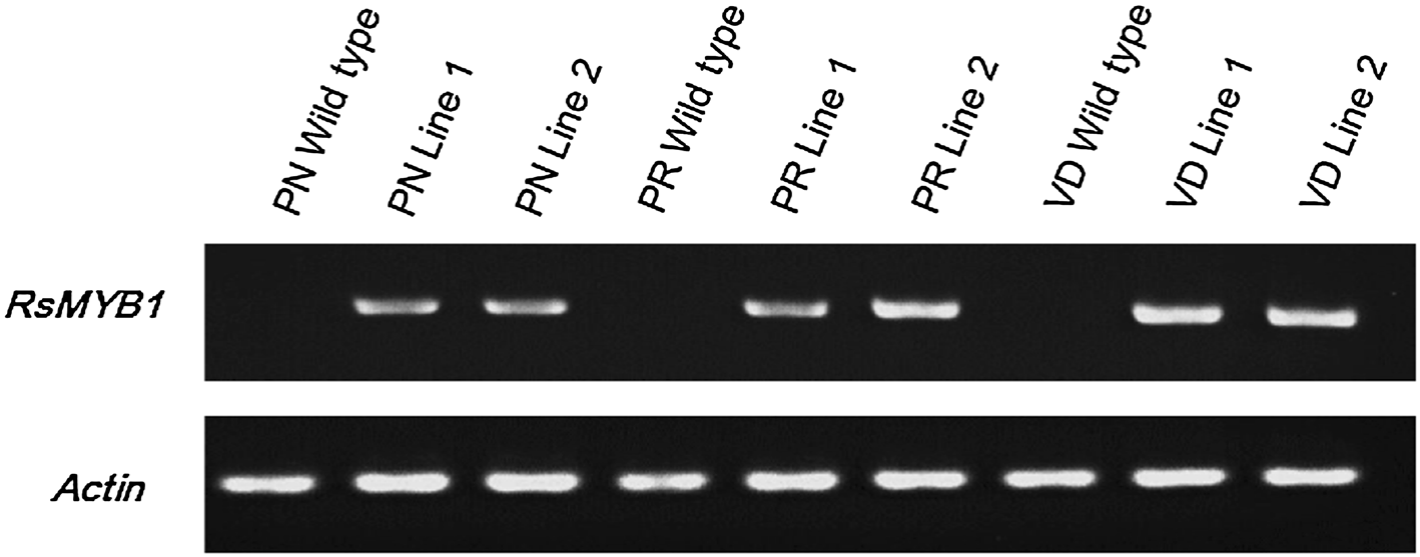
RT-PCR analysis of *RsMYB1* expression in the non-transgenic lines (wild type) and the transgenic lines of the chrysanthemum cultivars, namely, Peach ND (PN), Peach Red (PR), and Vivid Scarlet (VD)

We used the two independent transgenic lines derived from each cultivar to perform an expression analysis of *RsMYB1* and anthocyanin biosynthetic genes. Results of RT-PCR showed that distinct and stable expression of *RsMYB1* in all individual lines of the same genotype (Fig. 2), but its expression level was different along with different genotype tested. Surprisingly, distinct and stable expression of *RsMYB1* in the cultivars did not enhance anthocyanin production.

**Fig. 3:**
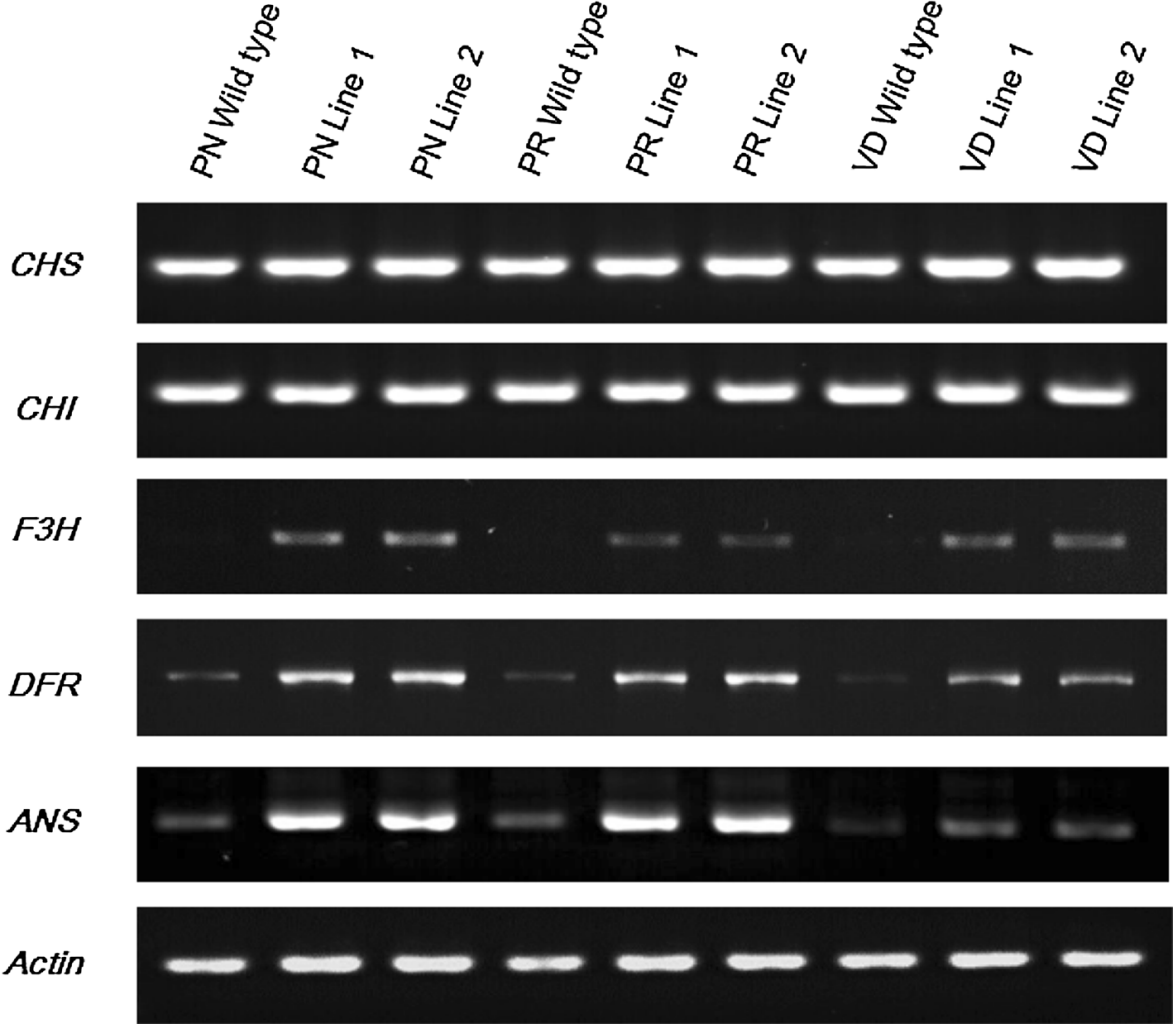
RT-PCR analysis of the expression patterns of biosynthetic genes in non-transgenic lines (wild type) and two individual transgenic lines of the chrysanthemum cultivars, namely, Peach ND (PN), Peach Red (PR), and Vivid Scarlet (VD) containing *RsMYB1*

Expression analysis of biosynthetic genes by RT-PCR revealed that the key biosynthetic genes (*CHI*, *CHS*, *F3H*, *DFR*, and *ANS*) were expressed in all transgenic lines, including the control. However, expression of *RsMYB1* in chrysanthemum could regulate the enhanced expression of the biosynthetic genes, in particular, *F3H*, *DFR*, and *ANS*, as compared to the expression of these genes in the control. Expression levels of other biosynthetic genes such as *CHI* and *CHS* did not differ between the controls and transgenic lines (Fig. 3). Interestingly, higher expression of *RsMYB1* is not associated with that of biosynthetic genes in the transgenic lines because expression levels of biosynthetic genes in ‘Peach ND’, which contains low transcripts of *RsMYB1*, were slightly higher than those in ‘Peach Red’ and ‘Vivid Scarlet’, which express high transcripts of *RsMYB1* (Figs. 2, 3).

### Analysis of anthocyanin content

**Fig. 4:**
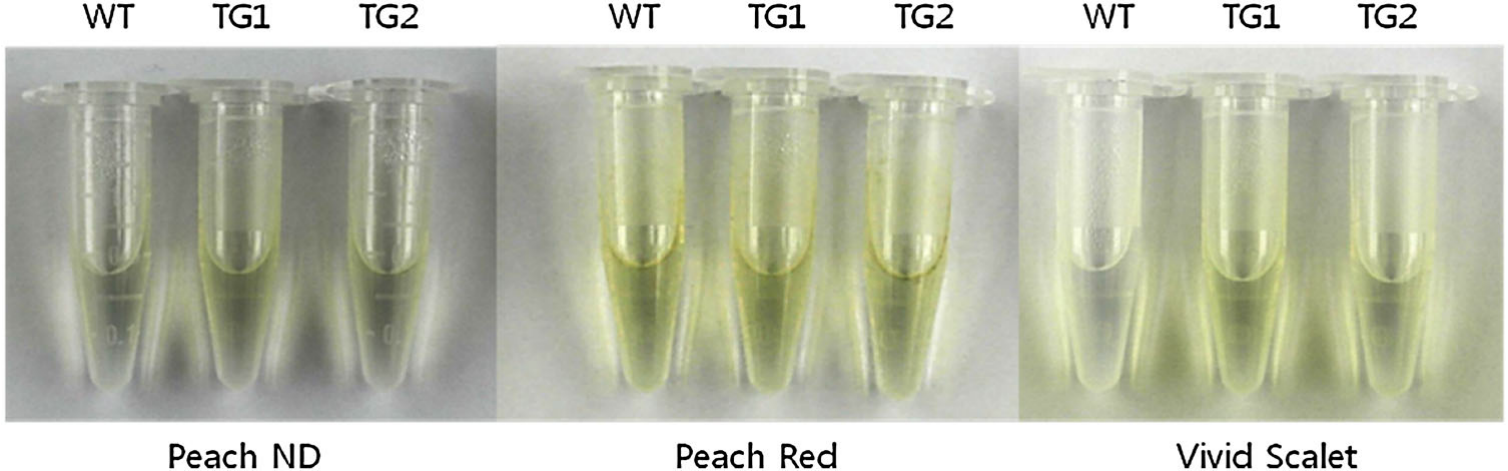
Comparative analysis of anthocyanin content between the non-transgenic lines (wild type) and transgenic lines of the chrysanthemum cultivars, namely, Peach ND (PN), Peach Red (PR), and Vivid Scarlet (VD) containing *RsMYB1*

**Fig. 5:**
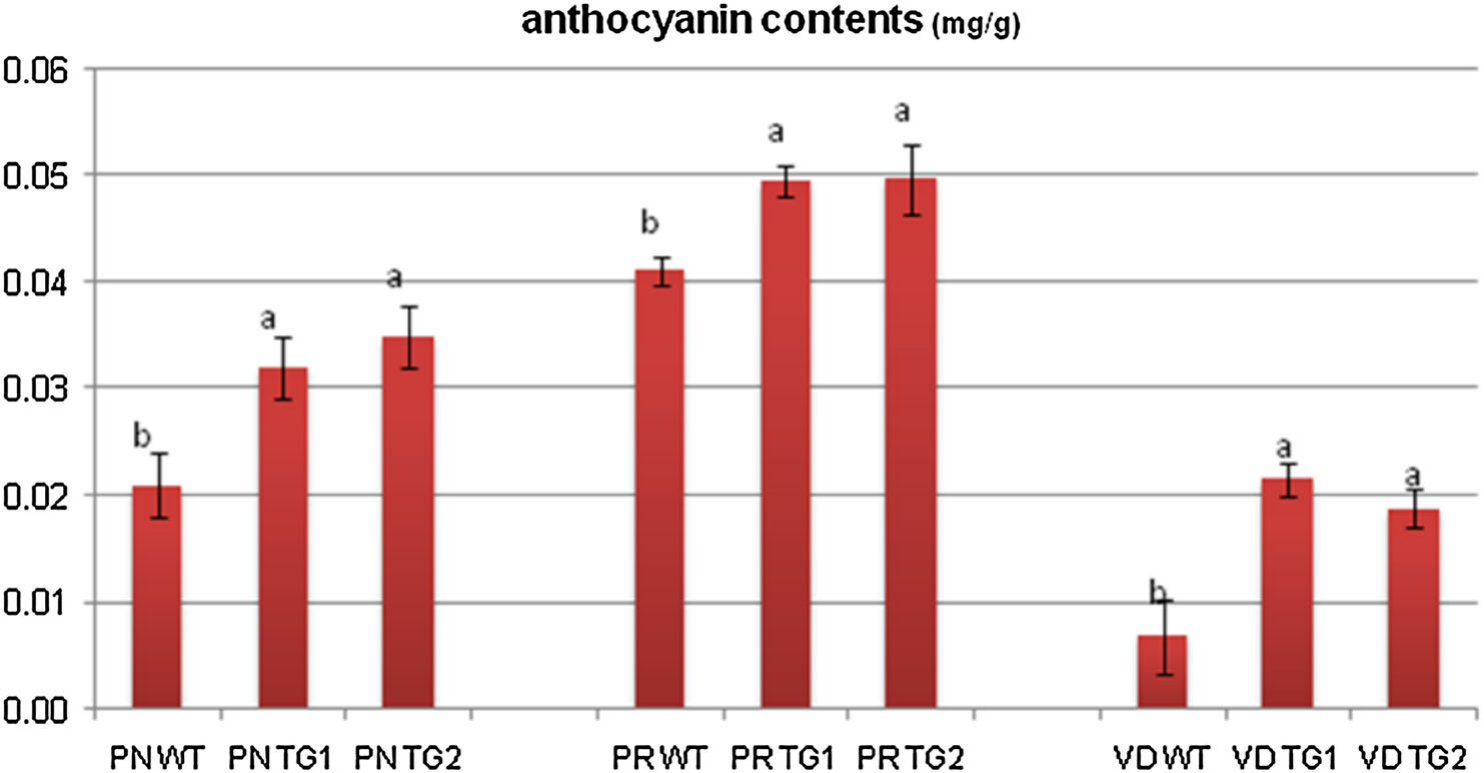
Spectrophotometeric analysis of anthocyanin content between the non-transgenic (wild type) and transgenic lines of the chrysanthemum cultivars, namely, Peach ND (PN), Peach Red (PR), and Vivid Scarlet (VD) containing *RsMYB1.* Bar stands for standard error. Means marked with same letter within the same cultivar are not significantly different

Although variations in biosynthetic gene expression were observed between the controls and transgenic lines (Fig. 3), enhanced anthocyanin accumulation was not visually observed in the transgenic lines. Hence, we assumed that anthocyanin might have accumulated inside the leaves without being expressed phenotypically. However, when anthocyanin was extracted from the leaves of the transgenic lines and compared to the extracts from the control plants, only slight significant color difference was observed in ‘Peach Red’ and ‘Peach ND’ while a distinct variation was seen in ‘Vivid Scarlet’ (Fig. 4). This was further confirmed using spectrophotometry results, which were not consistent with the results for anthocyanin color in (Fig. 5) in ‘Peach Red’, ‘Peach ND’ because anthocyanin contents were observed to be significantly different in the cultivars (Fig. 5), where the contents in transgenic lines were approximately 0.01–0.015 mg g^−1^ higher than those in the controls, regardless of genotypes. Thus, higher expression levels of the three biosynthetic genes were associated with anthocyanin accumulation in the cultivars.

## Discussion

Transcription factors can regulate genes involved in the anthocyanin biosynthetic pathway in various plants, including model plants and floricultural or food crops (Nishihara and Nakatsuka 2011). R2R3 MYB transcription factors, which regulate anthocyanin biosynthesis, cloned from petunia (Quattrocchio et al. 2006), apple, strawberry, plum and rose (Lin-Wang et al. 2010), lily (Yamagishi et al. 2010), snapdragon (Jackson et al. 1991), morning glory (Morita et al. 2006), gentian (Nakatsuka et al. 2008) and tobacco (Pattanaik et al. 2010) have been shown to regulate sets of genes in the anthocyanin biosynthetic pathway. Therefore, we assumed that it is possible to use the radish *RsMYB1* transcription factor, which belongs to the R2R3-MYB transcription factor family and was found to be highly expressed in the skin and flesh of three radish cultivars and responsible for anthocyanin accumulation (Park et al. 2011), to enhance anthocyanin biosynthesis in the chrysanthemum cultivars ‘Peach ND’, ‘Peach Red’, and ‘Vivid Scarlet’, which showed anthocyanin degradation under elevated temperatures.

Here, we produced two individual transgenic lines from each cultivar. However, not all shoots that showed positive results for the introduced *RsMYB1* exhibited anthocyanin-enhancing phenotypes. Butaye et al. (2005) claimed that this type of variation among individual transgenic plants/genotypes is often observed in plant transformation experiments and can be explained by variation in the expression of the introduced *RsMYB1*. Analysis of *RsMYB1* expression in the transgenic lines was shown to be stable using RT-PCR; this was not consistent with the claim of Butaye et al. (2005).

In addition, many researchers have documented that anthocyanin accumulation in chrysanthemum correlated with the expression pattern of biosynthetic genes (Chen et al. 2012; He et al. 2013; Sung et al. 2013). Since the main anthocyanin biosynthesis pathway in chrysanthemum is the cyanidin-based pathway (He et al. 2013), *CHS*, *CHI*, *F3H*, *DFR*, and *ANS* are considered as five key biosynthetic genes responsible for anthocyanin accumulation in chrysanthemum. Our results show that the expression of *RsMYB1* in chrysanthemum could enhance the expression level of key biosynthetic genes (*F3H*, *DFR*, and *ANS*), although anthocyanin accumulation is not visible in the cultivars. However, analysis of anthocyanin content using a spectrophotometer revealed that anthocyanin contents in transgenic lines of the cultivars were significantly higher than those in the controls. Thus, anthocyanin accumulation in the cultivars was correlated with the expression pattern of biosynthetic genes as reported by the researchers (Chen et al. 2012; He et al. 2013; Sung et al. 2013), but anthocyanin content produced by *RsMYB1* seemed to be genotype-dependent because enhancement of anthocyanin contents in cultivars ‘Peach ND’ and ‘Vivid Scarlet’ seemed to be higher than that in cultivar ‘Peach Red’ (Fig. 5). Quattrocchio et al. (1999) claimed that although MYB transcription factors could enhance anthocyanin production, different pigmentation control activities were observed in different species of *Antirrhinum* and *Petunia*. Moreover, different pigmentation effects of MYB transcription factors on berry skin color in grape (Kobayashi et al. 2004, 2005) and tuber color in potato (De Jong et al. 2004) have also been reported. There seems to be some variation in the ability of transcription factors to control anthocyanin production in different genotypes.

Color pigmentation in petunia due to *RsMYB1* was visually shown in our preliminary experiment (data not shown). In addition, enhancement of anthocyanin contents in the cultivars by *RsMYB1* was observed in this study. However, the reason why increased anthocyanin content was not visually observed in the cultivars is unclear. One possible explanation is that anthocyanin contents induced by *RsMYB1* was not dramatic in these cultivars. Another possible explanation is that the expression levels of *F3H*, *DFR*, and *ANS* were not high enough to enhance high anthocyanin content. Otherwise, it might be that expression of MYB transcription factor alone is not able to enhance strong anthocyanin accumulation in chrysanthemum, because the expression of MYB transcription factor alone failed to express anthocyanin in Arabidopsis and tobacco (Lloyd et al. 1992), and grape (Hichri et al. 2011). Alternatively, it also might be due to lack of basic helix–loop–helix (bHLH) expression in the chrysanthemums because, in several other cases, co-expression of MYB transcription factor and a bHLH partner are required to strongly express anthocyanin (Butelli et al. 2008; Lloyd et al. 1992; Quattrocchio et al. 1998). Therefore, we will conduct further experiments to reveal the reason why increased anthocyanin was not visually observed in the transgenic chrysanthemums expressing *RsMYB1*.

## Conclusions

We produced transgenic chrysanthemums that expressed *RsMYB1* and demonstrated the effects of its ectopic expression on the expression levels of anthocyanin biosynthetic genes. This study revealed that *RsMYB1* could enhance the expression of the biosynthetic genes (*F3H*, *DFR*, and *ANS*) in transgenic lines of the cultivars Peach ND, Peach Red, and Vivid Scarlet and increase anthocyanin contents in the cultivars. The reason for the lack of visual enhancement in anthocyanin contents is still unknown. Accordingly, further studies on promoter functions and environmental conditions that affect the anthocyanin production pathway are necessary to understand the regulatory mechanism underlying anthocyanin biosynthesis. Overall, our study provides important information regarding the role of *RsMYB1* in the regulation of key anthocyanin biosynthetic genes in chrysanthemums.
